# Decoding the Tumour Microenvironment: Molecular Players, Pathways, and Therapeutic Targets in Cancer Treatment

**DOI:** 10.3390/cancers16030626

**Published:** 2024-01-31

**Authors:** Eleonora Malavasi, Manuel Adamo, Elisa Zamprogno, Viviana Vella, Georgios Giamas, Teresa Gagliano

**Affiliations:** 1 Cancer Cell Signalling Laboratory, Department of Medicine, University of Udine, 33100 Udine, Italy; malavasi.eleonora@spes.uniud.it (E.M.); adamo.manuel@spes.uniud.it (M.A.); zamprogno.elisa@spes.uniud.it (E.Z.); 2 School of Life Sciences, University of Sussex, Brighton BN1 9QG, UK; vv62@sussex.ac.uk

**Keywords:** tumour microenvironment, cancer, epigenetics, kinases, phosphatases, metabolism, cytokines, hormones

## Abstract

**Simple Summary:**

Tumour cells are not independent entities but are always surrounded by different types of non-tumoural cells and other extracellular components, which together constitute a complex association known as the tumour microenvironment. Cancer cells and non-tumoural cells can influence each other in several ways that contribute to sustaining tumour growth and development. This complex crosstalk is mediated by a plethora of different molecules and pathways that, in the context of cancer, are dysregulated and altered. In this work, we reviewed the latest update regarding some of the molecules and pathways involved in the tumour microenvironment and their role in mediating tumour progression.

**Abstract:**

The tumour microenvironment (TME) is a complex and constantly evolving collection of cells and extracellular components. Cancer cells and the surrounding environment influence each other through different types of processes. Characteristics of the TME include abnormal vasculature, altered extracellular matrix, cancer-associated fibroblast and macrophages, immune cells, and secreted factors. Within these components, several molecules and pathways are altered and take part in the support of the tumour. Epigenetic regulation, kinases, phosphatases, metabolic regulators, and hormones are some of the players that influence and contribute to shaping the tumour and the TME. All these characteristics contribute significantly to cancer progression, metastasis, and immune escape, and may be the target for new approaches for cancer treatment.

## 1. Introduction

It is well known that a tumour is not an independent entity, but it is instead influenced by the surrounding environment, giving rise to the so-called tumour microenvironment (TME). This heterogeneous collection is composed of different types of cells, including immune and stromal cells, secreted factors, blood vessels, and the extracellular matrix; in its complexity, it is in continuous evolution [1]. The relationship between the tumour and the components of the TME is dynamic and reciprocal; it starts early in tumour development and sustains cancer cell survival, immune escaping, local invasion, and metastasis [2]. The TME and tumoural cells regulate and influence each other through the involvement of different processes, such as signal transduction, and paracrine and autocrine signals [3]. In addition, the TME sustains the tumour by providing oxygen through angiogenesis, nutrient supply, and eliminating metabolic waste. Cancer cells themselves are constantly evolving, resulting in the tumour being constituted by a heterogeneous population [4]; this is also true for the components of the TME, such as immune cells and myeloid cells [5,6]. In addition, the tumour microenvironment is specific and varies for each cancer, as each tumour involves a different organ [7]. 

Inside the tumour microenvironment, there are different populations of T cells that promote tumourigenesis and are specific for tumour-associated antigens. Non-physiological antigens expressed by tumours are detected by cytotoxic T cells (CD8+), which target cancer cells for destruction [8]. For these reasons, the presence of CD8+ cells in the TME of cancer patients is usually correlated with a positive prognosis [9]. Also, the presence of CD4+ T cells contributes to a positive outcome. Indeed, proinflammatory CD4+ T cells, T helper 1 (Th-1), sustain CD8+ cells with the production of interleukin-2 (IL-2) and IFN- γ [10,11]. Regulatory T cells (Tregs), on the other hand, are normally responsible for the suppression of the inflammatory response, and in the context of the TME, they promote tumour progression by sustaining it with the secretion of growth factors [12]. 

Also, B cells are important for the process of tumourigenesis. Indeed, they present a dualistic role. They have an anti-tumourigenic role by promoting cytotoxic immune response through antibody production and the secretion of cytokines, but they can also support tumour progression thanks to the production of other cytokines, which lead to an immune-suppressive phenotype [13]. 

Macrophages are one of the most abundant infiltrated cell types in the tumour microenvironment, which in some cases can represent up to 50% of a tumour’s mass [14]. Macrophages can be polarised in two forms that display different functions: M1 macrophages, which are pro-inflammatory and inhibit tumour progression, and M2 macrophages, which are anti-inflammatory and promote tumour growth [15]. Macrophages infiltrated in the tumour microenvironment are known as tumour-associated macrophages (TAMs) and are usually M2-like cells [16]. They were found to secrete vascular endothelial growth factor (VEGF) near the vessels of the tumour, and their presence in the TME is associated with disease progression and poor patient prognosis [17,18].

Dendritic cells (DCs) have a key role in the adaptive immune response and they are responsible for cross-priming tumour-specific T cells. Generally, their presence in the TME induces T cell response with the consequent reduction in cancer progression [19]. DCs infiltrated in the TME can present different maturation stages and the majority of tumours contain only a low number of mature DCs [20]. Clinical studies have shown that, in the tumoural context, DCs can present an altered differentiation and activation state, impairing anti-tumour immune response [21,22].

Cancer-associated fibroblasts (CAFs) are activated fibroblasts present in the tumour microenvironment, which display high heterogeneity and play a key role in regulating the biology of tumours. Indeed, they secrete different types of factors that influence tumour development, metastasis, and drug resistance [23]. 

**Figure 1:**
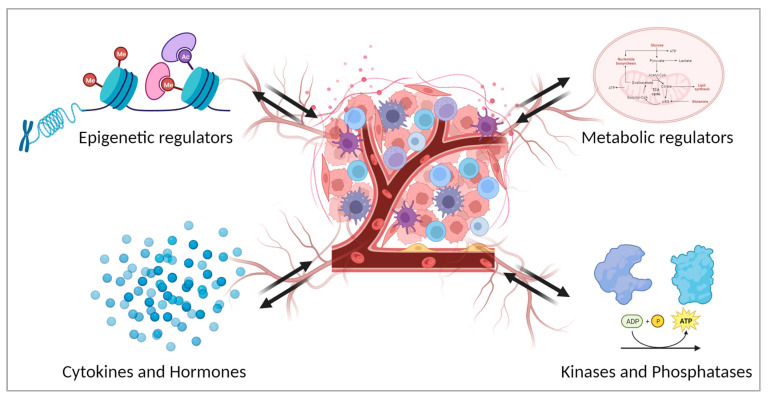
Different players that contribute to the formation of the tumour microenvironment and their reciprocal influence on tumour cells. Created with BioRender.com.

In the context of the tumour microenvironment, factors provided by this altered context also play a major role in the epithelial–mesenchymal transition (EMT) [24]. EMT is a multistep event where epithelial cells acquire mesenchymal properties losing epithelial characteristics. In TME the high production of inflammatory cytokines together with ROS determines the activation of potent EMT inducers [25]. As a result, activation of the EMT program in cancer cells enhances invasion and metastasis. In this review, we address the fundamental interplay and crosstalk between the TME and the tumour, focusing on the mechanisms that affect tumour development and progression. We will focus on different types of regulation, with particular attention on epigenetic and metabolic regulations, cytokines and hormones, kinases, and phosphatases (Figure 1). 

## 2. Epigenetic Regulators

Epigenetics is essential in several mechanisms and comprises changes in the gene expression that do not involve alterations in the DNA sequence; instead, it involves all the modifications that alter the chromatin packing [26]. Hence, these changes are more flexible and versatile, and can also propagate in various cell cycles [27]. Epigenetic programming is critical for the development and maintenance of tissue- and cell-type-specific functions [28]. Different epigenetic players, such as DNA methyltransferases (DNMTs) and histone-modifying enzymes, establish several interlocking mechanisms of epigenetic layers to maintain the stability of a locus in a particular active state. Indeed, the transcriptional status of most somatic cell genes is epigenetically controlled. Consequently, perturbation of this balance could give rise to gene expression alterations, causing malignant cell transformation [28]. 

Covalent modifications of histone tails include acetylation, methylation, phosphorylation, ADP-ribosylation, ubiquitination, and SUMOylation. The equilibrium of acetylation is maintained by the opposite action of HATs (histone acetyltransferases) and HDACs (histone deacetylases), which control not only the acetylation status of histones, but also that of many chromatin modifiers, transcriptional regulators, and intracellular signal transducers [26]. Both HATs and HDACs have been found to be dysregulated or mutated in different cancers. Indeed, HDACs present a dualistic role: many tumour-suppressor pathways depend on the HDAC function to exert their job, but on the other hand, HDACs play a significant role in the transcriptional inactivation of tumour-suppressor genes [28]. Also, DNA methylation presents a dualistic role, and it is strongly correlated with cancer development. General genomic hypomethylation influences oncogene expressions, such as CYCLIN D2 [29], BCL2 [30], and HRAS [31]; however, local hypermethylation of tumour-suppressor genes promotes oncogenesis [32]. In several human tumours, all three methyltransferases involved during early embryogenesis (DNMT1, DNMT3, DNMT3B) have been found to be overexpressed [33,34,35,36].

The role of the microenvironment in tumour development is increasingly evident, and epigenetic alterations affect it, influencing the initiation and progression of the tumour. Epigenetic machinery, together with the coordination of extracellular stimuli, signalling pathways, and specific transcription factors, influence myeloid differentiation and activation [37]. Indeed, myeloid cells present a high degree of plasticity during physiological and tumoural conditions, thanks to epigenetic mechanisms [38]. 

**Figure 2:**
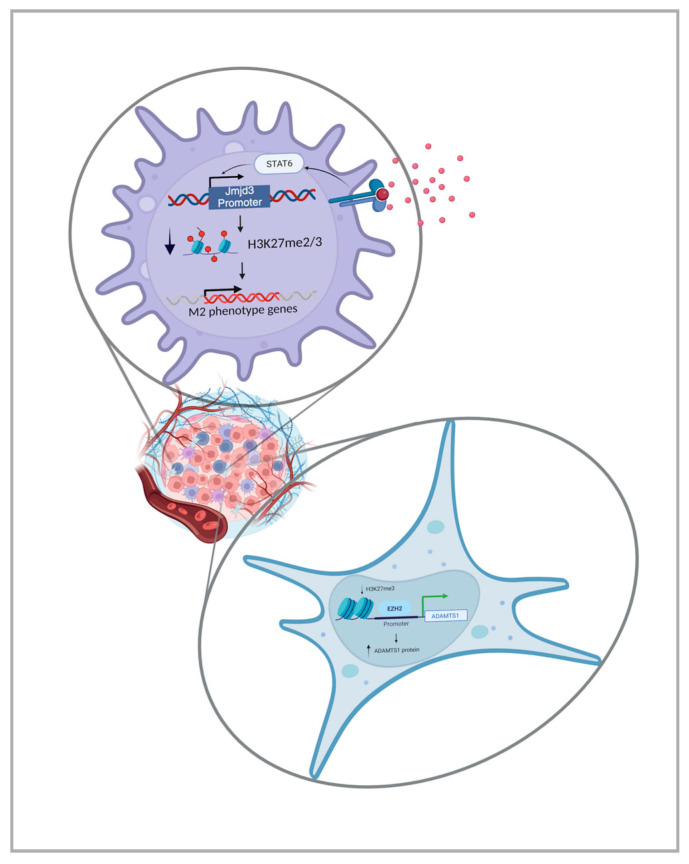
Epigenetic regulation mediated by the tumour microenvironment. In tumour-associated macrophages (purple), a decrease in H3K27me2/me3 is induced by Jmjd3 demethylase, which is activated upon the STAT6 signal after IL4 stimulation. In cancer-associated fibroblasts, hypomethylation at the promoter region of ADAMTS1, mediated by EZH2, determines the increased expression of the metallopeptidase. Created with BioRender.com.

One of the most abundant populations of leukocytes infiltrated in tumours are macrophages, named tumour-associated macrophages (TAMs); normally, they are associated with poor prognosis [39]. Macrophages are typically defined as M1 or M2. M1 macrophages secrete proinflammatory cytokines, such as IL1, IL6, IL12, and TNFα, and they exert anti-tumoural properties, stimulating the immune system. M2 macrophages, on the other hand, secrete anti-inflammatory cytokines, such as IL10 and TGFβ, display immunosuppressive behaviours, and deplete nutrients for T cell response against the tumour [40]. The TAM polarization from M1 to M2 within the tumoural environment might be strongly influenced by epigenetic machinery [41], as well as the switch from immunogenic DCs to immunosuppressive DCs [42]. For instance, HDAC inhibition affects DC differentiation and function by impairing CD1a, CD80, and CD83 expression, generating a less immunogenic phenotype [43]. In addition, IL-4 is responsible for the demethylation of a specific set of genes involved in DC differentiation, through a JAK3-STAT6 and TET2 pathway, linking cytokine-mediated events with innate immune cell differentiation [44]. Furthermore, IL-4 stimulation leads to H3K27 Jmjd3 demethylase expression through the STAT6 signal, inducing a decrease in H3K27me2/me3 and the transcriptional activation of M2-marker genes (Figure 2) [45]. 

In the context of the TME, the tumour affects myeloid lineage differentiation, which undergoes a pathological switch from canonical myeloid cells toward pathologically activated immature cells, named myeloid-derived suppressor cells (MDSCs). MDSCs include a variety of heterogeneous immature, immunosuppressive, and pathologically activated myeloid cells, which support the formation of the metastatic niche and tumour growth through angiogenesis and invasion [46]. The significant involvement of epigenetic mechanisms in the expansion and function of MDSCs is highlighted in several studies. Indeed, the pharmacological inhibition of HDACs enhances MDSC proliferation, in vitro and in vivo, promoting the role of acetylation during myeloid cell differentiation [47]. 

Cancer-associated fibroblasts (CAFs), myofibroblasts, stellate cells, and mesenchymal stromal cells (MSCs) constitute the functional and structural support of the tumour in the TME [48]. The lack of mutations in these cells in ovarian and breast carcinomas [49], which present an aberrant phenotype, suggests the involvement of epigenetic mechanisms in their contribution to tumour progression. In recent studies on gastric and lung cancer [50,51], as well as acute myeloid leukaemia [52], researchers have described a widespread DNA hypomethylation associated with the focal gain of DNA hypermethylation in CAFs compared to control fibroblasts, which had a general impact on gene expression. There are several targeted genes in cancer-associated MSCs involved in the hypermethylation process, such as SMAD3 [51], FRZB [53], PTPN6 [54], and SOCS1 [55], as well as genes targeted for hypomethylation. One example of hypomethylation is the reduction of EZH2-mediated H3K27me3 at the promoter region of ADAMTS1 in breast cancer, which determines the increased expression of the metallopeptidase with the consequent promotion of metastasis (Figure 2) [56]. Metastasis formation, which implies the transformation from an in situ to an invasive tumour, is one of the most critical events in cancer progression. It is well known that during this process, myoepithelial (ME) cells play a central role in the transition for invasion. Indeed, the basement membrane and ME cell layer are disrupted, allowing the invasion of tumour epithelial cells into adjacent tissues [57]. Another metalloprotease was found to be overexpressed in CAFs, ADAM12, a regulator of cell–matrix and cell–cell interactions; it has been involved in tumour progression [58]. Aberrant hypomethylation at the gene promoter region is the mechanism that mediates the overexpression of ADAM12, leading to an association with worse tumour growth outcomes in different types of cancer, like triple-negative breast cancer (TNBC) [59] and gastric cancer (GC) [60].

MiRNAs are involved in many fundamental cellular processes as they are estimated to control more than 50% of all protein-coding genes in mammals [61]. As a result, they have been linked to the regulation of processes that stimulate the development of cancer or, conversely, processes that may stop the development of cancer. For instance, a cancer cell can occur after the overexpression of so-called “onco-miRs”, which downregulate tumour suppressors that regulate cell proliferation (e.g., the miR-17-92 family, miR-21, and -155). On the other hand, miRNAs that target cellular oncoproteins, such as let-7 family members, miR-15a, -16, and -29, which act as tumour suppressors, are often downregulated in cancer tissues [62]. Different types of micro-RNA have been found to be epigenetically regulated in many types of cancer, including ovarian [63], gastric [64], and colorectal [65]. In addition, their overexpression can be correlated with poor prognosis, such as for miR-21 in colon cancer [66]. Within the TME, IL-6 and prostaglandin E2 (PGE2) play a crucial role in the crosstalk between cancer and stromal cells. miR-149 targets IL-6 mRNA and is downregulated in gastric cancer CAFs, resulting in the inhibition of fibroblast activation. This downregulation of miR-149 in CAFs is determined by hypermethylation of its promoter region and may contribute to the CAF phenotype [67]. Also, in breast cancer, different microRNAs are epigenetically regulated and influence tumourigenesis and the immune environment. Specifically, EZH2, which catalyses di- and trimethylation H3K27, determines the epigenetic silencing of miR-29b or miR-30d with the consequent promotion of LOXL4, which is positively correlated with macrophage infiltration [68]. 

From a broader perspective, all this evidence highlights the key role of epigenetics in cancer development. The influence of epigenetics is present in all cellular components of the tumour microenvironment, and the TME might be considered an epigenetic modifier. For this reason, epigenetic therapies represent new emerging approaches for cancer treatment, where the regulation of epigenetic mechanisms in different stromal and immune cells may be used to reshape the TME from being immunosuppressive to being anti-tumoural.

## 3. Kinases and Phosphatases

Protein phosphorylation is a regulation mechanism that is extremely important for cellular processes [69]. When considering the importance of phosphorylation in all key cellular processes, it is believed that protein kinases and phosphatases play a critical role in the different mechanisms associated with the tumour microenvironment, such as angiogenesis, tumourigenesis, metastasis, and therapy resistance [70].

**Figure 3:**
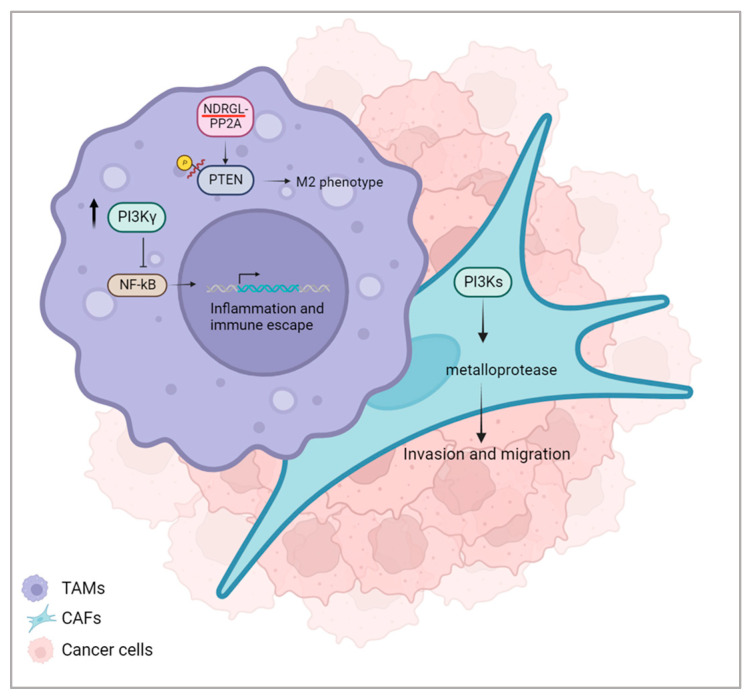
Some of the kinases and phosphatases involved in the TME-tumour crosstalk. In tumour-associated macrophages, altered activities of PI3Kγ and NDRGL-PP2 determine, respectively, the activation of inflammation transcriptional programs and the switch to the M2 phenotype. In cancer-associated fibroblasts, PI3Ks influence metalloprotease secretion, leading to a consequent increase in invasion and migration. Created with BioRender.com.

Most kinases target both serine and threonine (serine/threonine kinases, STKs), others target tyrosine (tyrosine kinases, TKs), and some act on all three (dual-specific kinases, DSKs) [71]. Phosphoinositide 3-kinases (PI3Ks) are another family of kinases involved in proliferation and survival [72]. In human cancer, the pathway of Class I PI3K is often disrupted by a series of mutations, which increase PI3K activity [73], and this has an impact on many mechanisms within the tumour microenvironment [74]. During tumour growth, macrophage PI3Kγ plays a crucial role in the switch toward immune suppression. Indeed, the inhibition of NF-kB induced by PI3Kγ signalling through Akt and mTor leads to the activation of transcriptional programs for inflammation and immune escape (Figure 3) [75]. Selective inhibition of PI3Kγ, highly expressed in myeloid cells, restores the sensitivity of tumours enriched with tumour-associated myeloid cells to immune checkpoint blocking (ICB) antibodies [76]. Furthermore, the inhibition of PI3Kδ in regulatory T cells and CD8+ T cells determines tumour regression [77]. PI3Ks also influence the activity of fibroblasts; they affect the matrix metalloprotease secretion of fibroblasts, which is critical in tumour cell invasion and migration during metastasis formation (Figure 3) [78].

Another important kinase is the Lyn kinase; it is involved in proliferation, migration, metabolism, and apoptosis, and it also has a well-established role in most hematopoietic cells [79]. Lyn kinases are essential for the microenvironment that supports chronic lymphocytic leukaemia (CLL) progression and growth. Indeed, their loss of function reduces B cell receptor signalling, including Burton tyrosine kinase (BRT) phosphorylation. In addition to this, Lyn-deficient macrophages fail to provide the necessary support for CLL cell survival [80]. 

Also, MAPKs are essential kinases involved in many cellular pathways. In tumour-associated macrophages, their ROS-mediated activation upregulates IL-12 with the activation of ERK1/2 and the consequent production of INF-γ by TAMs, and this can convert the macrophage phenotype toward the pro-immunogenic type [81]. 

Protein phosphatases (PPs) that regulate mammalian cellular processes are very different and there are at least six families of serine/threonine PP that exert the majority of protein phosphatase activity in several cell types [82]. It is well known that hypoxic conditions in the TME sustain tumour progression and survival [83], and the principal hypoxia mediator is hypoxia-inducible factor 1 alpha (HIF1α) [84]. It has been shown that, under hypoxia conditions, protein phosphatase type 1 (PP1), a serine/threonine phosphatase involved in the dephosphorylation of several proteins, is inhibited through its association with the nuclear inhibitor of PP1 (NIPP1). Consequently, a decrease in cAMP/PKA-dependent signalling occurs, an event that may play a role in the adaptation of cells to hypoxic conditions [85]. Also, PP2A, another serine/threonine PP, takes part in the hypoxia signalling pathway. In human aortic smooth muscle cells (HASMCs) and AC16 cells, the hypoxic status decreases the PP2A mRNA expression and protein activity in HIF1α-dependent and -independent manners [86]. Protein phosphatases also play a crucial role in the microenvironment that regulates hepatocellular carcinoma (HCC). Indeed, intracellular phosphorylation signalling pathways involved in cellular maintenance can respond to stimulation in the extracellular environment [87]. PTEN is fundamental for macrophage polarization and function in the TME. Through the NDRG2-PP2A complex, PTEN is dephosphorylated and activated, and this results in the promotion of cancer progression by increasing the number of M2 TAMs (Figure 3) [88]. Imbalances in kinases and phosphatases in the tumour microenvironment are key players in tumour development and growth. For this reason, different inhibitors that target these enzymes have been used to counteract disease progression [89]. Unfortunately, different types of tumours still cannot rely on proper treatment options; consequently, it is important to find new drugs that inhibit kinases and phosphatases to enhance the efficacy of standard therapeutic approaches. 

## 4. Metabolic Regulators

**Figure 4:**
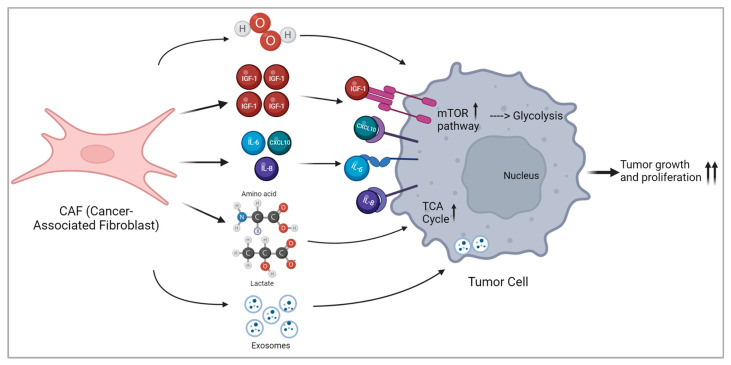
Metabolic regulators that are involved in the crosstalk between CAFs and tumour cells in the context of the tumour microenvironment. Created with BioRender.com.

Metabolism is a process involving a network of biochemical reactions that convert nutrients into small molecules called metabolites [90]. Through these resulting metabolites, cells generate energy, redox equivalents, and macromolecules required for survival [90]. Under normal conditions, cells derive energy by initially engaging it through glycolysis within the cytosol, followed by mitochondrial oxidative phosphorylation (OXPHOS) when oxygen is available (if oxygen is limited, cells opt for glycolysis instead of the oxygen-dependent mitochondrial metabolism to meet their energy requirements). However, the metabolic pattern of tumours is different from that of normal cells [90]. Indeed, metabolic reprogramming of tumour cells is essential for the initiation, proliferation, and progression of cancer [91]. This is a cancer hallmark that endows cancer cells with growth and proliferative potential in the nutrient-poor tumour microenvironment [92]. Recent studies have demonstrated that TME is characterised by a hypoxic and acid environment with electrolyte imbalance and elevated oxidative stress [91,92]; it is important in shaping the metabolic landscape of a tumour (Figure 4) [91]. Otto Warburg was the first to observe that tumours consume glucose and secrete lactate regardless of the availability of oxygen [91,92] (this is usually called the “Warburg effect” or “aerobic glycolysis” [90]). In particular, cancer cells can upload glucose thanks to the upregulation of glucose transporters 1 and 3 (GLUT1 and GLUT3), in order to provide the precursor and intermediate metabolites and produce a high amount of lactate [93]. The collateral metabolic fluxes, arising from the Warburg effect, lead to the activation of a specific pathway, such as the pentose phosphate pathway (PPP) and one-carbon pathway [93].

Because the infinite proliferation of tumour cells requires a faster energy supply, the ATP production rate of glycolysis is much faster than oxidative phosphorylation, although it has a lower efficiency in terms of ATP production per molecule of glucose [90]. To support cancer cell energy demand or anabolic processes, these cells use other core metabolic processes, such as glutaminolysis and fatty acid oxidation [92]. The metabolic abnormalities that characterise tumour cells are not simple alterations of a metabolic pathway but are rather subversive alterations in the entire cellular network metabolism [94]. In addition, due to the high metabolic activity of cancer cells, metabolic reprogramming has also been reported to occur in the TME; this is considered one of the hallmarks of cancer [94].

As stated above, TME is a highly complex and heterogeneous ecosystem, with peculiar structural and biophysical characteristics [93], which include tumour cells, immunosuppressive cells (as tumour-associated mesenchymal stem cells (TA-MSCs), CAFs (which are abundant and critical for the TME [95]), myeloid-suppressor cells (MSCs), immune and inflammatory cells, intercellular stroma, microvasculature, and biomolecules infiltrating from nearby regions [96]. The characteristics of the neoplastic tissue are effectively conveyed through intricate interactions involving cancer and stromal cells, coordinated by soluble compounds, metabolites, extracellular vesicles (EVs), and direct cell-to-cell communication [93]. Emerging evidence indicates that cancer cells are capable of suppressing anti-tumour immune responses by depleting and competing for essential nutrients or reducing the metabolic fitness of tumour-infiltrating immune cells [90]. As a consequence of this immune response, a dramatic modification in tissue metabolism occurs, involving the depletion of nutrients, the increase of oxygen consumption, and the generation of reactive oxygen species and intermediates. The aberrant metabolites or intermediates of cancer metabolism could have a crucial role in regulating the differentiation, proliferation, activation, and function of immune cells [90]. The harsh TME and oncogenic background are responsible for the increase in reactive oxygen species (ROS) in tumour cells. To challenge the toxic levels of ROS, tumours increase their antioxidant capacity to allow cancer progression (PPP activation is oriented in this way) [93].

Stromal cells in the TME contribute to ECM remodelling, migration, invasion, and evasion of immunosurveillance, and these cellular processes are sustained by cellular metabolism and local nutrient composition [91]. Tumour-associated stromal cells are derived from different cell types, which give origin to cancer-associated fibroblasts, cancer-associated adipocytes (CAAs), or cancer-associated endothelial cells (CAECs) [91]. As mentioned before, CAFs are one of the most abundant cell populations in TME [91] and promote tumour progression via the secretion of various growth factors, cytokines, chemokines, H_2_O_2_, and the degradation of ECM [95]. They can also affect cancer cell growth via their metabolic pathways [95]. The crosstalk between CAFs and tumour cells is often referred to as a “reverse Warburg effect” because metabolites secreted from CAFs (lactate and pyruvate [97]) are utilised as fuel for neighbouring tumour cells [91]. In this model, cancer cells use oxidative stress in order to extract nutrients from stromal cells to cope with the nutrient-poor microenvironment, secreting hydrogen peroxide or miRNAs (for this reason, they are called “metabolic parasites”) [98]. 

Cancer-associated fibroblasts employ aerobic glycolysis, leading to the secretion of lactate, which in turn can support the metabolism of cancer cells. They can also increase glutamine anabolic metabolism (glutamine is secreted in TME and is consumed by cancer cells to sustain nucleotide generation and OXPHOS) [91]. CAFs can secrete aspartate (which is useful for nucleotide biosynthesis and proliferation in multiple tumours), and CAF-derived exosomes have been found to provide amino acids, lipids, and TCA intermediates in pancreatic and prostate cancer, to sustain central metabolism [91]. 

In PDAC, stroma-associated pancreatic stellate cells (a cell population very similar to activated CAFs [93]) can secrete alanine, which is uploaded by pancreatic cancer cells and fuels their TCA cycle, leading to the increased biosynthesis of lipids and non-essential amino-acids [94]. They can also secrete lysophosphatidylcholine, which supports the production of phosphatidylcholine, which in turn supports the membrane synthesis and production of LPA, allowing PDAC growth and migration [91].

Adipose stromal cells secrete arginine, which can be consumed by ovarian and endometrial cancer cells and converted into citrulline (which enhances adipogenesis) and nitric oxide (NO, which reduces oxidative stress and promotes glycolysis) [91]. In particular, omentum ASCs (ASCs that are contained in the omentum) increase NO synthesis with the consequent boost in glycolysis in cancer cells, and this increase in NO has an inhibitory role on the enzymes involved in mitochondrial respiration. This occurs through the s-nitrosylation of hexokinase, a key player in the initial stage of glycolysis, which is highly expressed in cancer cells and converts glucose to glucose-6P [99]. In low oxygen conditions, characterised by low NO concentrations, HIF1a (hypoxia-inducible factor 1-a) orchestrates the shift in energy metabolism from oxidative phosphorylation to glycolysis by regulating glucose transporter-1 (GLUT-1), lactate dehydrogenase (LDH), and pyruvate dehydrogenase (PDH) expression [99]. In addition, it has been demonstrated that O-ASCs positively influence the Warburg effect by modulating the NO homeostasis [99]. In cancer cells O-ASCs secrete arginine, which enhances NO synthesis, resulting in the reshaping of the metabolic profile by increasing glycolysis and decreasing mitochondrial ATP generation [99]. Cells in ovarian and endometrial cancer use arginine produced by O-ASCs to generate citrulline, which can determine an increase in the adipogenesis of O-ASCs [99]. 

## 5. Cytokines and Hormones

Neoplastic cells and the elements involved in the tumour microenvironment interact to produce pro- and anti-tumour signals. Direct cell-to-cell communication or secreted chemicals, including growth factors, cytokines, chemokines, and microRNAs, are possible ways that the tumour and stroma can communicate with one another [100]. In the early phases of tumour development, immune cells and the soluble substances they secrete create a specific microenvironment that favours anticancer activities; nevertheless, as the microenvironment changes, the immune cells are modified to support tumour growth [101]. Therefore, the tumour microenvironment is substantially larger than the neoplasm's primary unit. Its complexity results from the multidirectional and dynamic interactions among resident or recruited elements, ultimately resulting in the loss of normal tissue architecture, inflammatory sites, hypoxia, and neoangiogenesis, taking into account the high demands for oxygen and nutrients [102].

Among growth factors, TGF-β causes anti-proliferative reactions in a variety of cell types, including both transformed and normal epithelial cells; interfering with the factor’s signalling might play a role in the pathophysiology of cancer [103]. TGF-β signalling may still be important at later stages of tumour growth if the function of TGF-β—as an inhibitor of epithelial tumourigenesis—needs to be reduced. On the other hand, compared to the normal surrounding tissue, tumour cells have elevated TGF-β expression, most frequently TGF-β1, and release TGF-β ligands. Additionally, increased TGF-β expression is linked to tumour growth and a poor prognosis, suggesting that TGF-β has a pro-cancer function in later stages of the disease [104].

The complex network of cells that make up the immune system includes NK cells, which are innate lymphoid cells (ILCs), and NKT cells, macrophages, and dendritic cells, which are phagocytic mononuclear system cells involved in antigen presentation. B lymphocytes and T lymphocytes, including CD4+ and CD8+ T cells, are part of the adaptive immune response. Activated NK cells secrete soluble mediators such as tumour necrosis factor-alpha (TNF-a), interferon-gamma (IFN-g), interleukin (IL)-10, chemokines like CCL3, CCL4, CCL5, XCL1, and others, as well as growth factors like granulocyte–macrophage colony-stimulating factor (GM-CSF) [105]. CD4+ T cells are important immune system regulators that can develop into several T-helper cell lineages: T helper 1 (TH1) cells that support cell-mediated immunity and T helper 2 (TH2) cells that support humoral immune responses produce interferon and interleukin 4 (IL-4), respectively [39]. Through the expansion of the cytotoxic CD8+ T-cell (CTC) population, TH1 and TH2 cells can improve anti-tumour immunity. By blocking cytotoxic T cells, regulatory T (Treg) cells, on the other hand, reduce anti-tumour immunity. IL-17 is secreted by TH17 cells. In contrast to TH1 cells, which are largely anti-tumour, TH2 cells actively polarise tumour-associated macrophages (TAMs) to advance cancer [106]. Depletion of Tregs promotes tumour growth, as demonstrated by Noy and Pollard’s research: CD4+ Tregs are immune-suppressive, directly reducing the ability of CD8+ cytotoxic T cells to fight tumours by secreting IL-10 and transforming growth factor β. In the presence of transforming growth factor-β, IL-6, and IL-1, CD4+ T cells become TH17 cells. The ability of CD4+ TH17 cells to play pro-tumourigenic or anti-tumourigenic functions in tumour immunity and inflammation relies on the stimuli they encounter [107].

Inflammatory interleukins from the same family that are present in the tumour might either stimulate or inhibit immune responses to it. Most cytokines mediate a greater pro-tumourigenic effect in colorectal cancer (CRC). For instance, the pro-inflammatory cytokine IL-6 suppresses apoptosis and the generation of reactive oxygen species (ROS) in cells like monocytes and macrophages. This cytokine, together with IL-10, IL-11, and IL-23, serves as an alarm to reduce inflammation when the body is in a homeostatic state [108]. Other alarm cytokines, such as IL-1α and IL-1β, are released by pathogen-recognition receptors in response to DAMPs and PAMPs, and they initiate and amplify local inflammation. While IL-18, another member of the IL-1 family of cytokines, is dramatically lowered in CRC patients, IL-1α and IL-1β are significantly elevated, indicating a significant anti-tumourigenic effect in colorectal cancer. One cytokine from this family, IL-24, was identified as having tumour-preventing properties [109]. Greater expression of the chemokine CCL-2 causes CRC to have a poor prognosis, whereas higher expression of the same chemokine causes breast cancer to have a favourable prognosis [110]. The most studied ligand, CCL16, has demonstrated an anti-cancer impact in mouse colon carcinoma and breast cancer through enhanced expression [111], attributable to an increase in CD4+ T cells, CD8+ T cells, and DC infiltration into the tumour, as well as in prostate cancer.

Due to their important roles in malignancies, CCL2 and CLL7 have also been thoroughly studied as therapeutic targets in cancer therapy. Promising results have been obtained using CCL2 siRNA, CCR2 siRNA, CCL2-neutralizing antibodies, CCL2 inhibitors, or CCR2 antagonists to treat tumours like breast cancer [112], glioma [113], and hepatocellular cancer [114] in laboratory animals that received cancer cell implantation.

Type I IFNs have several potential effects on tumour growth, including inhibiting proliferation and angiogenesis, activating innate cells and adaptive immune response, and bridging innate and adaptive immunity [115]. In tumours, as a response to DNA fragments, IFNα and IFNβ are secreted by cancer cells and DCs, resulting in the activation of the cGAS/STING pathway with the consequent T cell priming and anti-tumour activity [116]. IFNγ is mainly produced by T cells and NK cells in response to a variety of inflammatory or immune stimuli. Within the tumour, tumour-infiltrating lymphocytes are the main source of IFNγ, which have displayed particular importance in tumour immunosurveillance [117]. Lactate acidosis is one of the factors that can regulate IFNγ expression in tumour-infiltrating NK cells and T cells, which negatively regulates IFNγ production by NK cells in the context of tumour transformation [118]. IFNγ decreases tumour cell growth in different ways, such as through apoptosis and necroptosis, and by inducing tumour cell cycle arrest, enhancing expression of the cell cycle inhibitor proteins p27Kip, p16, or p21 in different cancers, like breast cancer [119] and hepatocellular cancer [120]. Through the induction of mitochondrial-derived ROS, which is dependent on cytosolic phospholipase A2 (cPLA2) activation, IFNγ induces autophagy-associated apoptosis in colorectal cell lines [121]. On the other hand, IFNγ triggers an increase in the STAT1-dependent miR-29a/b, but not cell cycle inhibitor proteins, in melanoma cell lines, and there is a negative relationship between miR-29a/b expression and the proliferation rate of different cell lines [62].

## 6. Conclusions

In conclusion, the tumour microenvironment is a very complex collection of cells, molecules, extracellular components, and processes, which are altered and contribute to the sustenance of the tumour. In this review, we focused on epigenetic regulation, kinases, phosphatases, metabolic regulators, cytokines, and hormones, but many other players are involved in the process of carcinogenesis. Further studies are required to better understand the complex interplay between cancer cells and the TME and to find new therapeutic strategies against tumours.
